# Why cancer incidence in the Arab counties is much lower than other parts of the world?

**DOI:** 10.1186/s43046-022-00142-3

**Published:** 2022-10-03

**Authors:** Mostafa A. Arafa, Karim H. Farhat

**Affiliations:** 1 Cancer Research Chair, College of Medicine, King Saud University, Riyadh, 11922 Saudi Arabia; 2 Community Medicine Department, High Institute of Public Health, Alexandria University, Alexandria, Egypt

**Keywords:** Cancer, Incidence, Middle East, Arabs

## Abstract

Despite the relatively increased cancer incidence in the last few years in the Arab countries, it is still far from the figures reported from Western countries. Several mechanisms have been adopted to explain the significant decreased incidence of cancer in the Arab countries, among them fasting, food full of special recipes filled with spices, significant lower rates of smoking and alcohol drinking, and genetic predisposition. Clinical trials are warranted on a large population scales to study, discuss, and verify such mechanisms.

## Background

Cancer is one of the prominent causes of death worldwide, killing about 10 million people in 2020 [1]. Most common (for new cancer cases) were breast (2.26 million cases), lung (2.21 million cases), colon and rectum (1.93 million cases), prostate (1.41 million cases), skin (non-melanoma) (1.20 million cases), and stomach cancers (1.09 million cases). Cancer is growing at an alarming pace in the Middle East region countries. Long-term projections show that, by 2030, there would be a 1.8-fold increase in cancer incidence [2]. Yet, the incidence is very low in comparison to Western countries and the USA [3, 4].

Among the top 10 countries reporting the highest cancer rates for men and women combined, Australia sits at the highest, with 452.4 cases reported per 100,000 people, using an age-standardized method. New Zealand follows with 422.9 cases by 100,000 people. The USA ranked the fourth [5]. On the other hand, the reported figures from Middle East countries are much lower. The highest number of cases was Egypt and Lebanon, 159.4 and 165.8 cases by 100,000 people respectively; the lowest number was reported from Saudi Arabia and Sudan, 96.4 and 95.7 respectively (Table 1).

**Table 1:** Estimated age-standardized incidence rates (World) in 2020, all cancers, both sexes, all ages

Arab countries		Developed countries	
Country	Value	Country	Value
Egypt	159.4	Australia	452.4
Lebanon	156.8	New Zealand	422.9
Jordan	155.3	Ireland	372.8
Morocco	148.3	United States of America	362.2
Algeria	135.3	Denmark	351.1
Iraq	134.9	The Netherlands	349.6
Tunisia	133.5	Belgium	349.2
Libya	132.2	Canada	348
Kuwait	115.7	France	341.9
Bahrain	112.2	Hungary	338.2
Qatar	107.2	Norway	327.5
United Arab Emirates	106.7	United Kingdom	319.9
Oman	103.8	Switzerland	317.6
Saudi Arabia	96.4	France, New Caledonia	313.7
Sudan	95.7	Germany	313.2

Source: Cancer Today—Global Cancer Observatory—IARC 2020

## Main text

Such lower figures of cancer in the Middle East, Arab countries could be attributed to several factors and theories:

I- Fasting

Fasting Ramadan is a religious rite that extends over a course of 1 month, where Moslems refrain from eating and drinking from dawn to dusk, which is intermittent fasting, and questions have been raised about its potential role in the prevention and treatment of cancer. The general theory behind intermittent fasting in cancer is the difference in how cells adapt to stress. It is believed that healthy cells can adapt much better to less nutrients in the environment. On the other hand, cancer cells continue to grow, increasing the need for nutrients. In 2016, a study investigated the role of long-term nighttime fasting in breast cancer recurrence 36% more likely to have breast cancer recurrence than those who fasted at night 13 h or more [6]. The study of Southern California University suggests that fasting may enhance the sensitivity of cancer cells to chemotherapy and may guard normal cells and promote stem cell production [7].

Onodera et al .hypothesized that tiny molecular agents, which may preferentially reduce neoplastic cell survival under nutrient-deprived conditions, could function as anticancer drugs. In his study, they constructed a high-throughput screening system to spot such small molecules and screened chemical libraries and microbial culture extracts. They were able to determine that some small molecular compounds, like penicillic acid, papyracillic acid, and auranofin, exhibit preferential cytotoxicity to human carcinoma cells under nutrient-deprived compared with nutrient-sufficient conditions [8].

Until we have human studies indicating a benefit from intermittent fasting, it is important to examine the potential mechanism: how cancer might be affected by intermittent fasting. It has been suggested that this may support the role of timed/long-term fasting in the prevention or treatment of cancer.

a- Decreased inflammation

Many studies suggest the role of inflammation in cancer development and the progression and spread of existing cancer. It is well known that inflammatory markers in the blood are associated with a poor prognosis chronic inflammation as well as cancer can interfere with the treatment of cancer [9]. A study in 2019 found that intermittent fasting can reduce inflammation. After a short fast, both monocyte counts and inflammatory activity decreased [10].

b- Enhancement of immunity

Fasting induces changes associated with cellular protection to actually protect against weight loss initially and increases protection from oxidative stress. Fasting results in a more significant drop in insulin levels, along with an upsurge in insulin sensitivity in a shorter amount of time. Given that insulin levels play a role in cancer risk, these differences are potentially clinically important [11]. In 2014, London et al. showed that fasting in mice killed “old” immune cells and replaced them with stem cells when subjects began eating again. They concluded that a 3-day fast could help regenerate a strong immune system [12].

c- Autophagy

Autophagy is an evolutionarily conserved lysosomal catabolic technique with the aid of using which cells eighty three degrade and recycle intracellular endogenous and exogenous additives to maintain mobile homeostasis [13].

The role of autophagy in cancer is complex and its function can vary from one to another: biological factors such as tumor type, stage of progression, and genetic status; oncogene activation and tumor suppressor inactivation, thus autophagy may be associated; and to prevent tumor formation or to enable adaptation, proliferation, survival, and metastasis of cancer cells [14]. Protocols aimed at inducing autophagy rather than blocking it are currently being intensively studied in oncology [15, 16]

d- Slowing cancer growth and promoting cell regeneration

Fasting might also additionally lessen glucose levels within the blood, making it tougher for cancers to grow. Cancer cells feed on glucose, eating it at a far better degree than ordinary cells do. During fasting, one manner cells attempt to preserve strength is with the aid of using making it less complicated for cell membranes to metabolize insulin. This improves insulin’s capacity to get rid of glucose from the blood. Reducing the glucose to be had to most cancers cells might also additionally restrict cancerous growth.

e- Promoting cell regeneration

An important question is whether the body could be utilized from fasting through replacing the unneeded cells and other damaged parts during a process called autophagy. Studies have shown that lack of autophagy reduces tumor suppressor gene levels. One way to activate autophagy is fasting, which puts stress on the body’s cells. Turn on autophagy to make cells function more efficiently [17].

II- Spices

Arab people in the East Mediterranean region are using a lot of different spices in their food which have an antioxidant and anti-cancer effects:

a- Turmeric: Turmeric reduces inflammation, which is at the root of many diseases, including cancer. Animal and lab studies show that turmeric can help prevent cancer growth and kill certain cancer cells. Small studies among people with cancer show that turmeric can help improve quality of life [18]. A phase 2 study that combined curcumin and conventional chemotherapy to treat people with advanced colorectal cancer is still ongoing [19].
b- Cinnamon: Impaired apoptosis plays an important role in the development and progression of cancers. Increased evidence suggests that cinnamon as a therapeutic agent has an anti-cancer effect by affecting a number of apoptosis-related pathways in cancer cells [20].
c- Cardamom: The compounds in cardamom may help fight cancer cells. Studies in mice have shown that cardamom powder can increase the activity of certain enzymes that help fight cancer. The spice may also enhance the ability of natural killer cells to attack tumors [21, 22].
d- Saffron: It has selective toxicity against cancer cells, through inhibition of (ribonucleic acid) RNA and (deoxyribonucleic acid) DNA synthesis and increasing apoptosis. Crocin is considered the most important anticancer drug in saffron, which plays an important role in gene expression and apoptosis in cancer cells [23].
e- Nutmeg, black seed, dill, and sesame seed: Nutmeg compounds inhibits some aspects of the metabolism of cancer cells, by killing malignant cells while preserving normal and healthy cells cell. The compounds contained in nutmeg suppress inflammation by blocking molecular processes such as nitric oxide synthase [24]. Black cumin (*Nigella sativa*) (NS) is native to the Mediterranean and neighboring countries Pakistan and India. It is included in the lifestyle and daily diet as a spice and preservative. Black seeds have been used for thousands of years to treat a variety of illnesses and illnesses. Over the last 50 years, many scientific studies have confirmed and demonstrated the pharmacological quality of NS seeds. Its anti-inflammatory, antibacterial, antihistamine, and anticancer [25].

III- Alcohol and cigarette smoking

It is well documented that alcohol and tobacco smoking are significantly associated with many types of cancers, particularly lung cancer and colorectal cancer. The percentage of females who are smoking in the Middle East is significantly lower in comparison to Western countries; in addition, alcohol is prohibited in many Islamic countries in the Middle East. The highest percentage of smoking among females was reported from France (33%), Hungary, and Germany (26%); the corresponding rates in Egypt, Kuwait, Algeria, Morocco, Saudi Arabia, and Qatar were 0.4, 2.5, 0.8, 0.9, 2.1, and 2 respectively. The highest rate was reported from Lebanon 28.7% [26].

IV- Genetic predisposition

Although the rapid change in incidence rates of breast cancer in Middle East and North Africa (MENA) countries, the young age at onset and high grade suggest the influence of genetic factors such as mutation of breast cancer 1 (BRCA1) and breast cancer 2 (BRCA2) genes [27]. Hereditary breast and ovarian cancers originate from BRCA1 and/or BRCA2 gene mutations that significantly increase the likelihood of developing breast, ovarian, prostate, and other types of cancer [28]; however, BRCA mutations are not as prevalent among Arab breast cancer patients as they are among other ethnic groups [29].

## Conclusions

In the Middle East, we have witnessed a surprisingly accelerated incidence of some cancers, which are often secondary to adverse lifestyle choices and westernization. Despite this, the incidence is far lower that reported figures from Western countries. Several clinical trials are warranted to study the effect of caloric restriction, fasting, and type of food in the Middle East on cancer incidence. There is a high need to trace hereditary cancer genes across the Arab world.

## Data Availability

Not applicable
